# Association between body composite indices and vertebral fractures in pre and postmenopausal women in Korea

**DOI:** 10.1371/journal.pone.0254755

**Published:** 2021-08-04

**Authors:** HyunJin Kim, Chung-woo Lee, Myung Ji Nam, Yeon Joo Choi, Kyungdo Han, Jin-Hyung Jung, Do-Hoon Kim, Joo-Hyun Park

**Affiliations:** 1 Department of Family Medicine, Korea University Ansan Hospital, College of Medicine, Korea University, Ansan-si, Republic of Korea; 2 Department of Statistics and Actuarial Science, Soongsil University, Seoul, Republic of Korea; 3 Department of Biostatistics, College of Medicine, The Catholic University of Korea, Seoul, Republic of Korea; Universidad Nacional Autonoma de Mexico, MEXICO

## Abstract

The association between obesity and vertebral fracture remains controversial. This study aimed to investigate the association between obesity/abdominal obesity and vertebral fracture according to menopausal status. This nationwide population-based epidemiologic study collected data from the Korean National Health Insurance Services to investigate the association between obesity/abdominal obesity and vertebral fracture in pre and postmenopausal women who underwent national cancer screening in 2009. We used three body composite indices of obesity, body mass index, waist circumference and waist-to-height ratio, to classify participants into obesity and abdominal obesity groups. In both pre and postmenopausal groups, participants with obesity showed a higher risk of vertebral fracture and the association was stronger in those with abdominal obesity (*p* < 0.001). Participants with obesity showed a high risk of vertebral fracture, and the association was stronger in participants with abdominal obesity (*p* < 0.001). In both pre and postmenopausal groups, participants with obesity showed a higher risk of vertebral fracture (adjusted HR, 1.24; 95% CI, 1.19–1.30), (adjusted HR, 1.04; 95% CI, 1.03–1.05, and those with abdominal obesity showed even higher risk of vertebral fractures (adjusted HR, 1.35; 95% CI, 1.27–1.43), (adjusted HR, 1.13; 95% CI, 1.11–1.14). Vertebral fracture risk is higher in pre and postmenopausal women with obesity and even higher in those with abdominal obesity. Therefore, weight management can prevent vertebral fractures.

## Introduction

Menopause is a normal yet very crucial event in women’s life since it causes several changes that can influence women’s health. The average age at menopause is 49.3 years in Korean women; therefore, women spend 40% of their life in the postmenopausal state. It is well established that menopause is a known cause of bone loss and can deteriorate bone health [1]. The Study of Women’s Health Across the Nation (SWAN), which is a large, multi-ethnic cohort study of women across the United States, has shown that early bone loss in menopause is caused by the alteration in estradiol and follicle-stimulating hormone levels during menopause [2]. Fractures are a major health problem among older people, particularly in older women, and can result in significant morbidity and mortality [3]. Therefore, it is important to identify the determinants that affect bone health to prevent further bone loss and architectural damage caused by menopause in subsequent years.

Obesity is also a common public health problem. For many years, the association between obesity and bone health has been studied. Previous studies on the association between obesity and bone health have commonly examined body mass index (BMI) and bone mineral density (BMD). The Rancho Bernardo study enrolled 1 492 participants aged 55 to 84 and found a correlation between weight and BMD [4]. However, the effect of obesity on fracture risk remains controversial. Although high BMD cannot necessarily be equal to better bone health, numerous studies have shown that high BMI is associated with increased BMD. This is because the high mechanical load and endocrine effect of adipose tissue have a protective effect on bone density [5, 6]. However, recent studies have shown that obesity is negatively associated with bone mass [7, 8], and fractures in postmenopausal women with obesity contribute significantly to the overall fracture burden. The inconsistencies between these results were attributed to specific populations, backgrounds, and methodological differences.

Recent data have shown that the association between BMI and fracture risk varies according to the fracture site. For instance, the risk of hip fracture and wrist fracture was lower in postmenopausal women with obesity [9–11]. In contrast, the risk of ankle fracture and leg fracture was higher in postmenopausal women with obesity, and the results of such studies were consistent [12, 13]. However, studies on the relationship between obesity and vertebral fractures showed conflicting results. This discrepancy may have arisen from the different body compositions of different populations. Vertebral fractures are the most frequent fragility fractures and affect morbidity and mortality in older people [14]. In addition, vertebral fractures are associated with reduced quality of life and an increased risk of refracture at the same site and fractures in the future [3, 15].

BMI is the most widely used parameter for measuring obesity. However, BMI has limitations regarding the ability to discriminate lean mass from fat mass. Moreover, a study has shown a higher risk for osteoporosis in the low BMI category, which was thought to be related to sarcopenic obesity with low muscle mass [16] addition, BMI is influenced by both height and weight, and they independently contribute to the fracture risk. Therefore, in this study, we added additional indices to measure obesity such as waist circumference (WC) and waist-to-height ratio (WHtR). Recent studies have indicated that WHtR can be used as a substitute to measure the correlation matrix between BMI and WC owing to its ability to measure central obesity and predict health risk [17, 18].

Thus, we aimed to evaluate the association between obesity and the risk of vertebral fractures using three different body composite indices of obesity among pre and postmenopausal women using a nationwide population-based epidemiologic study.

## Materials and methods

### Study design and population

Data were collected from the Korean National Health Insurance Services (KNHIS) database in 2009, which is a nationwide database containing medical information that represents approximately 97% of the population in Korea. The KNHIS database includes data on age, sex, diagnosis, health check-up results, and drug prescriptions. From this database, we extracted information on women aged 30 and older who have received national cancer screening program and answered a questionnaire on their medical history including their reproductive factors. Diagnoses were coded according to the International Classification of Diseases 10^th^ Revision (ICD-10). The KNHIS database covers the data of 100% of the population, except for data on cosmetic surgeries and traffic or industrial accidents. Therefore, it has the advantage of focusing on non-traumatic fractures.

Among 3 280 834 women aged >30 years who underwent cancer screening in 2009, those who did not answer the questionnaire on reproductive factors and those who reached menopause after hysterectomy were excluded. Women who experienced menarche at <5 years and >30 years and women who experienced menopause at <30 years and >70 years were excluded. After excluding subjects with missing data and subjects with past fractures, 2 524 179 subjects were finally selected.

Access to these data was approved by the Korea University Ansan Hospital Institutional Review Board (IRB no. 2019AS0117), and the data were used after board review.

### Definition of fracture group

We identified patients with new-onset fracture using the ICD-10 and used the S12.0 (fracture of first cervical vertebra), S12.1 (fracture of second cervical vertebra), S12.2 (fracture of other specified cervical vertebra), S22.0 (fracture of thoracic vertebra), S22.1 (Multiple fracture of thoracic spine), S32.0 (fracture of lumbar vertebra), M48.4 (fatigue fracture of vertebra), M48.5 (collapsed vertebra, not elsewhere specified) codes for identifying a diagnosis of fracture.

### Anthropometric measurements

Anthropometric indices of obesity and abdominal obesity, including BMI, WC, and WHtR, were measured. Height and weight were measured, and BMI was calculated by dividing the weight (kg) by the square of the height (m). We identified obese group with BMI ≥ 25 kg/m^2^ and participants were also categorized according to the World Health Organization criteria based on BMI: <18.5 kg/m^2^ (underweight), 18.5–23 (normal weight), 23–25 (overweight), 25–30 (obese), and > 30 kg/m^2^ (severely obese) [19].

WC was measured in the horizontal plane at the mid-point between the anterior iliac crest and the inferior margin of the rib. Women with a WC > 85 cm were diagnosed with abdominal obesity [20], and based on WC, women were categorized into five groups: < 65, 65–75, 75–85, 85–95, and ≥ 95 cm [20].

WHtR was calculated as the WC (cm) divided by the height (cm). Hsieh et al. proposed a cutoff value of 0.5, and a WHtR > 0.5 has been proven to be associated with a higher risk for cardiovascular risk in Japanese adults [21]. According to studies conducted in Asian countries, WHtR has been suggested as a useful measure of central obesity in Asian populations, and the proposed cutoff point of WHtR is approximately 0.5 [22–25]. Therefore, women with WHtR > 0.5 represented abdominal obesity group, and we also classified them into quartiles.

### Assessment of covariates

A health questionnaire was used to obtain information on age, sex, comorbidities, blood test results, lifestyle habits, reproductive factors including current menstrual status and use of hormone replacement therapy and oral contraceptives. According to the answers to the question of ‘Do you still experience menstrual periods?’ in the questionnaire, we classified subjects into pre and postmenopausal groups.

Trained medical technicians performed standardized procedures to measure the body weight (kg) and height (cm) of all participants. We identified diseases such as hypertension, dyslipidemia, chronic kidney disease (CKD), cancer, and anemia. Hypertension, dyslipidemia, and cancer were identified by those who answered “yes” to the question of having been diagnosed with the same. CKD was defined as a glomerular filtration rate < 60 ml/min/1.73m^2^. Monthly household income and education were used as the main indicators of socioeconomic status. Participants were asked to state their highest educational degree, and we identified those who were educated up to high school level or above. Household income was evaluated based on equivalent income classified into quintiles, and we identified those who were included in the lowest quintile. The participants were also classified based on the frequency of alcohol intake (more or less than once every month), smoking status (whether currently smoking or not), and level of physical activity (identified as those who answered “yes” to the question of performing regular exercise).

### Statistical analysis

The association between different obesity indices and vertebral fracture risk in the pre and postmenopausal groups was evaluated using a Cox proportional hazard model with covariate adjustment using propensity scores based on age, sex, smoking status, frequency of alcohol intake, household income, exercise, hypertension, dyslipidemia, and hormone replacement therapy. The confidence interval (CI) was set to 95%. All data were analyzed using the Statistical Analysis System, release 9.4 (SAS Inc., Cary, NC, USA).

## Results

### Baseline characteristics of the subjects

Table 1 shows the general characteristics in the premenopausal and postmenopausal groups. Among the 2 524 179 subjects, 1 156 174 were classified as premenopausal and 1 368 005 were classified as postmenopausal. Data for all subjects in both groups were analyzed. As expected, the postmenopausal group showed a higher proportion of subjects with comorbidities such as diabetes mellitus, hypertension, dyslipidemia, and depression. The average weight in the premenopausal group was 57.44±8.18 kg, and the average weight in the postmenopausal group was 57.06±8.29 kg; thus, the average weight was similar. However, in terms of BMI, the proportion of participants with obesity (BMI ≥ 25 kg/m^2^) was higher in the postmenopausal group, and the postmenopausal group showed a higher proportion of participants with abdominal obesity (*p* < 0.001) (Table 1).

**Table 1:** Baseline characteristics according to menopausal status.

	Menopausal status		
	Premenopause	Postmenopause	*p*-value
n	1156174	1368005	
Age group, years			< .0001
30–39	152 210 (13.16)	124 (0.01)	
40–49	837 079 (72.4)	48 358 (3.53)	
50–59	161 140 (13.94)	557 092 (40.72)	
60–69	4 228 (0.37)	488 969 (35.74)	
70–79	1 517 (0.13)	273 462 (19.99)	
Smoking status			< .0001
Non	1 092 180 (94.47)	1 316 461 (96.23)	
Ex-smoker	22 141 (1.92)	14 616 (1.07)	
Current	41 853 (3.62)	36 928 (2.7)	
Alcohol intake			< .0001
Non	813 761 (70.38)	1 199 137 (87.66)	
Mild	328 850 (28.44)	161 963 (11.84)	
Heavy	13 563 (1.17)	6 905 (0.5)	
Low income	257 377 (22.26)	261 222 (19.1)	< .0001
Regular physical exercise	189 921 (16.43)	250 959 (18.34)	< .0001
Rheumatic arthritis	25 347 (2.19)	72 584 (5.31)	< .0001
Hyperthyroidism	24 048 (2.08)	35 235 (2.58)	< .0001
Chronic kidney disease	332 (0.03)	1 075 (0.08)	< .0001
Chronic obstructive pulmonary disease	47 219 (4.08)	124 092 (9.07)	< .0001
Hypopituitarism	149 (0.01)	564 (0.04)	< .0001
Hyperparathyroidism	468 (0.04)	1 129 (0.08)	< .0001
Cushing syndrome	273 (0.02)	1 311(0.1)	< .0001
Hyperprolactinemia	1 572 (0.14)	381 (0.03)	< .0001
Vitamin D deficiency	166 (0.01)	599 (0.04)	< .0001
Idiopathic hypercalciuria	3 008 (0.26)	9 753 (0.71)	< .0001
Diabetes Mellitus	38 721 (3.35)	180 364 (13.18)	< .0001
Hypertension	143 833 (12.44)	588168 (42.99)	< .0001
Dyslipidemia	123 111 (10.65)	466 535 (34.1)	< .0001
Intestinal malabsorption	187 (0.02)	392 (0.03)	< .0001
Chronic liver disease	16 713 (1.45)	40 847 (2.99)	< .0001
Anorexia nervosa	130 (0.01)	542 (0.04)	< .0001
Systemic lupus erythematosus	1 378 (0.12)	1 833 (0.13)	0.001
Irritable bowel disease	1 473 (0.13)	2 730 (0.2)	< .0001
Secondary amenorrhea	8 402 (0.73)	1 080 (0.08)	< .0001
Convulsions	1 422 (0.12)	3 426 (0.25)	< .0001
Depression	35 131 (3.04)	95 163 (6.96)	< .0001
Use of tricyclic and tetracyclic antidepressants	50 (0)	57 (0)	0.8477
Panic disorder	2 317 (0.2)	3 016 (0.22)	0.0005
Anxiety disorder	74 033 (6.4)	193 855 (14.17)	< .0001
Use of bisphosphonates	3 877 (0.34)	126 326 (9.23)	< .0001
Chronic kidney disease	48 028 (4.15)	163 806 (11.97)	< .0001
Obesity	271 440 (23.48)	511 616 (37.4)	< .0001
Body mass index, kg/m^2^		< .0001	
<18.5		41 667 (3.6)	29632(2.17)
18.5–23	584 780 (50.58)	466 693 (34.11)	
23–25	258 287 (22.34)	360 064 (26.32)	
25–30	236 565 (20.46)	451 606 (33.01)	
≥30	34 875 (3.02)	60 010 (4.39)	
Abdominal obesity	130 598 (11.3)	383 498 (28.03)	< .0001
Age, years	43.81±5.38	61.56±8.42	< .0001
Height, cm	157.79±5.24	153.52±5.7	< .0001
Weight, kg	57.44±8.18	57.06±8.29	< .0001
Body mass index, kg/m^2^	23.07±3.11	24.19±3.16	< .0001
Waist circumference, cm	74.88±8.17	80.04±8.6	< .0001
Waist-to-height ratio	0.48±0.05	0.52±0.06	< .0001
Fasting plasma glucose, mg/dL	93.26±17.56	99.8±24.46	< .0001
Systolic BP, mm Hg	116.63±14.22	125.69±16.21	< .0001
Diastolic BP, mm Hg	72.79±9.91	76.93±10.18	< .0001
Total cholesterol, mg/dL	190.68±39.02	208.03±44.03	< .0001
Total cholesterol, mg/dL	190.68±39.02	208.03±44.03	< .0001
Total fractures	48 757 (4.22)	259 544 (18.97)	< .0001
Vertebral fracture	8 601 (0.74)	108 493 (7.93)	< .0001
Hip fracture	810 (0.07)	15 418 (1.13)	< .0001
Follow-up duration, years	9.14±1.06	8.35±2.39	< .0001

### Association between body composite indices of obesity and prevalence of vertebral fracture in pre and postmenopausal women

We used the Cox proportional hazards model to estimate the incidence of vertebral fracture according to the presence of obesity and abdominal obesity.

Subjects were divided into two groups according to the presence of obesity based on BMI. In the premenopausal group, women with obesity (BMI >25 kg/m^2^) showed a significantly high risk of vertebral fracture (adjusted hazard ratio [HR], 1.24; 95% CI, 1.19–1.30). In the postmenopausal group, women with obesity showed a high risk of vertebral fracture (adjusted HR, 1.04; 95% CI, 1.03–1.05) (Table 2).

**Table 2:** Cox proportional hazards regression analysis for the development of vertebral fracture according to obesity status based on body mass index in the premenopausal and postmenopausal groups.

Premenopause						
**Obesity**	**N**	**Event**	**Duration**	**Rate**	**Without adjustment**	With adjustment*
No	884 734	5 875	8 101 053.16	0.73	1 (Ref.)	1 (Ref.)
Yes	271 440	2 726	2 470 695.1	1.10	1.52 (1.46,1.59)	1.24 (1.19,1.3)
Postmenopause						
**Obesity**	**N**	**Event**	**Duration**	**Rate**	**Without adjustment**	With adjustment*
No	856 389	65 846	7 136 899.39	9.23	1 (Ref.)	1 (Ref.)
Yes	511 616	42 647	4 281 488.58	9.96	1.08 (1.07,1.09)	1.04 (1.03,1.05)

We analyzed the relationship between vertebral fracture and abdominal obesity based on both WC and WHtR. Table 3 shows that in the premenopausal group, women with abdominal obesity (WC > 85 cm) showed a high risk of vertebral fracture (adjusted HR, 1.35; 95% CI, 1.27–1.43), and in the postmenopausal group, women with abdominal obesity showed an increased risk of vertebral fracture (adjusted HR, 1.13; 95% CI, 1.11–1.14) (Table 3). Abdominal obesity defined by WHtR showed similar results. The risk of vertebral fracture was increased in premenopausal women (adjusted HR, 1.32; 95% CI, 1.26–1.38) and postmenopausal women (adjusted HR, 1.21; 95% CI, 1.19–1.23) with a WHtR > 0.5 (Table 4).

**Table 3:** Cox proportional hazards regression analysis for the development of vertebral fracture according to abdominal obesity status based on waist circumference in the premenopausal and postmenopausal groups.

Premenopause						
**Abdominal obesity**	**N**	**Event**	**Duration**	**Rate**	**Without adjustment**	With adjustment*
No	1 025 576	7 014	9 385 869.38	0.75	1 (Ref.)	1 (Ref.)
Yes	130 598	1 587	1 185 878.88	1.34	1.79 (1.7,1.89)	1.35 (1.27,1.43)
Postmenopause						
**Abdominal obesity**	**N**	**Event**	**Duration**	**Rate**	**Without adjustment**	With adjustment*
No	984 507	70 487	8 272 308.85	8.52	1 (Ref.)	1 (Ref.)
Yes	383 498	38 006	3 146 079.11	12.08	1.42 (1.4,1.44)	1.13 (1.11,1.14)

**Table 4:** Cox proportional hazards regression analysis for the development of vertebral fracture according to abdominal obesity status based on waist-to-height ratio in the premenopausal and postmenopausal groups.

Premenopause						
**Abdominal obesity**	**N**	**Event**	**Duration**	**Rate**	**Without adjustment**	With adjustment*
No	817 352	4 897	7 488 693.03	0.65	1 (Ref.)	1 (Ref.)
Yes	338 822	3 704	3 083 055.23	1.20	1.84 (1.76,1.92)	1.32 (1.26,1.38)
Postmenopause						
**Abdominal obesity**	**N**	**Event**	**Duration**	**Rate**	**Without adjustment**	With adjustment*
No	484 720	26 061	4 135 973.59	6.30	1 (Ref.)	1 (Ref.)
Yes	883 285	82 432	7 282 414.37	11.32	1.8 (1.77,1.82)	1.21 (1.19,1.23)

*Adjustment for age, smoking status, alcohol intake, income, regular exercise, diabetes mellitus, hypertension, dyslipidemia, rheumatic arthritis, hyperthyroidism, chronic kidney disease, chronic obstructive pulmonary disease, hypopituitarism, hyperparathyroidism, Cushing’s syndrome, hyperprolactinemia, vitamin D deficiency, hypercalciuria, intestinal malabsorption, chronic liver disease, anorexia nervosa, systemic lupus erythematosus, irritable bowel disease, secondary amenorrhea, convulsions, depression, use of tricyclic and tetracyclic antidepressants, panic disorder, anxiety disorder, and use of bisphosphonates.

### Association between different levels of each body composite indices of obesity and the prevalence of vertebral fracture in pre and postmenopausal women

Participants were categorized into five BMI levels and analyzed according to menopausal status. Premenopausal women showed a statistically significant association between BMI and risk of vertebral fracture, and this association remained significant even after adjustment for age, smoking, alcohol intake, exercise, medication, and comorbidities known to have an effect on bone health such as diabetes, hypertension, dyslipidemia, and chronic kidney disease. After adjustment, except in the underweight group with a BMI < 18.5 kg/m^2^, BMI and vertebral fracture were positively associated. This association was also seen in postmenopausal women, but it was stronger in premenopausal women (Table 5).

**Table 5:** Cox proportional hazards regression analysis for the development of vertebral fracture according to BMI in the premenopausal and postmenopausal groups.

Premenopause						
**BMI (kg/m**^**2**^**)**	**N**	**Event**	**Duration**	**Rate**	**Without adjustment**	With adjustment*
<18.5	41 667	212	382481.41	0.55	0.83 (0.72,0.96)	1.03 (0.90,1.185)
18.5–23	584 780	3 575	5359686.96	0.67	1 (Ref.)	1 (Ref.)
23–25	258 287	2 088	2358884.8	0.89	1.32 (1.26,1.40)	1.12 (1.06,1.18)
25–30	236 565	2 334	2154220.56	1.08	1.63 (1.54,1.71)	1.27 (1.21,1.34)
≥30	34 875	392	316474.54	1.24	1.86 (1.68,2.06)	1.5 (1.35,1.67)
Postmenopause						
**BMI (kg/m**^**2**^**)**	**N**	**Event**	**Duration**	**Rate**	**Without adjustment**	With adjustment*
<18.5	29 632	3 268	229 073.85	14.27	1.60 (1.54,1.66)	1.1 (1.06,1.14)
18.5–23	466 693	34 941	3 888 313.01	8.99	1 (Ref.)	1 (Ref.)
23–25	360 064	27 637	3 019 512.52	9.15	1.02 (1.002,1.03)	1.02 (1.00,1.04)
25–30	451 606	37 611	3 779 928.19	9.95	1.11 (1.09,1.12)	1.05 (1.03,1.07)
≥30	60 010	5 036	501 560.38	10.04	1.12 (1.08,1.15)	1.08 (1.05,1.11)

BMI, body mass index

Participants were also classified according to five WC levels. Both pre and postmenopausal groups showed a positive association between WC levels and the risk of vertebral fracture. However, the association was stronger in the premenopausal group, and postmenopausal women with WC < 65 cm showed a higher risk of vertebral fracture. (Table 6)

**Table 6:** Cox proportional hazards regression analysis for the development of vertebral fracture according to WC in the premenopausal and the postmenopausal groups.

Premenopause						
**WC (cm)**	**N**	**Event**	**Duration**	**Rate**	**Without adjustment**	With adjustment*
<65	78 401	364	720 700.24	0.51	0.79 (0.71,0.88)	0.95 (0.85,1.06)
65–75	529 325	3 092	4 852 182.83	0.64	1 (Ref.)	1 (Ref.)
75–85	417 850	3 558	3 812 986.32	0.93	1.47 (1.4,1.54)	1.21 (1.15,1.27)
85–95	110 679	1 287	1 006 167.97	1.28	2.01 (1.88,2.14)	1.44 (1.34,1.54)
≥95	19 919	300	179 710.91	1.67	2.63 (2.33,2.96)	1.78 (1.58,2.01)
Postmenopause						
**WC (cm)**	**N**	**Event**	**Duration**	**Rate**	**Without adjustment**	With adjustment*
<65	29 032	1 871	242 278.27	7.72	1.09 (1.04,1.15)	1.2 (0.97,1.07)
65–75	324 722	19 440	2 752 137.37	7.06	1 (Ref.)	1 (Ref.)
75–85	630 753	49 176	5 277 893.21	9.32	1.32 (1.3,1.34)	1.12 (1.11,1.14)
85–95	318 300	30 926	2 618 775.12	11.81	1.67 (1.64,1.71)	1.22 (1.19,1.24)
≥95	65 198	7 080	527 303.99	13.43	1.91 (1.86,1.96)	1.28 (1.25,1.32)

WC, waist circumference

In WHtR, we classified the participants into quartiles. Similar to the results of BMI and WC, WHtR was positively associated with the risk of vertebral fractures in both pre and postmenopausal women, and the association was stronger in the premenopausal group. (Table 7)

**Table 7:** Cox proportional hazards regression analysis for the development of vertebral fracture according to WHtR_Q in the premenopausal and the postmenopausal groups.

Premenopause						
**WHtR_Q**	**N**	**Event**	**Duration**	**Rate**	**Without adjustment**	With adjustment*
Q1	290 029	1 342	2 664 177.19	0.50	1 (Ref.)	1 (Ref.)
Q2	289 546	1 740	2 652 118.5	0.66	1.3 (1.21,1.4)	1.09 (1.01,1.17)
Q3	287 290	2 266	2 624 025.67	0.86	1.71 (1.6,1.83)	1.26 (1.18,1.35)
Q4	289 309	3 253	2 631 426.9	1.24	2.46 (2.3,2.62)	1.49 (1.39,1.59)
Postmenopause						
**WHtR_Q**	**N**	**Event**	**Duration**	**Rate**	**Without adjustment**	With adjustment*
Q1	342 168	16 934	2 929 014.51	5.78	1 (Ref.)	1 (Ref.)
Q2	341 647	23 456	2 881 366.93	8.14	1.41 (1.38,1.44)	1.15 (1.12,1.17)
Q3	342 646	30 291	2 843 514.96	10.65	1.85 (1.81,1.88)	1.26 (1.23,1.28)
Q4	341 544	37 812	2 764 491.56	13.68	2.37 (2.33,2.42)	1.31 (1.28,1.33)

WHtR_Q, waist-to-height ratio quartiles; Q, quartile

*Adjustment for age, smoking status, alcohol intake, income, regular exercise, diabetes mellitus, hypertension, dyslipidemia, rheumatic arthritis, hyperthyroidism, chronic kidney disease, chronic obstructive pulmonary disease, hypopituitarism, hyperparathyroidism, Cushing’s syndrome, hyperprolactinemia, vitamin D deficiency, hypercalciuria, intestinal malabsorption, chronic liver disease, anorexia nervosa, systemic lupus erythematosus, irritable bowel disease, secondary amenorrhea, convulsions, depression, use of tricyclic and tetracyclic antidepressants, panic disorder, anxiety disorder, and use of bisphosphonates.

### Association between the composition of obesity and abdominal obesity, and the prevalence of vertebral fracture in pre and postmenopausal women

Participants were categorized into four composite groups based on two factors: obesity, defined as a BMI >25 kg/m^2^, and abdominal obesity, defined as a WC >85 cm.

In premenopausal women, with a group without obesity and abdominal obesity as a reference, participants with obesity but without abdominal obesity showed an increased risk of vertebral fracture. Furthermore, participants who had abdominal obesity with or without obesity also showed an increased risk of vertebral fracture, which was higher than the risk of the group without abdominal obesity. In postmenopausal women, participants with obesity but no abdominal obesity showed a lower risk of vertebral fracture (adjusted HR, 0.98; 95% CI, 0.96–0.999). Two composite groups with abdominal obesity, irrespective of the presence of obesity, showed a higher risk of vertebral fracture (Table 8).

**Table 8:** Composite study on obesity and abdominal obesity according to BMI and WC using Cox proportional hazards regression analysis for the development of vertebral fracture in the premenopausal and postmenopausal groups.

Premenopause							
**BMI**	**WC**	**N**	**Event**	**Duration**	**Rate**	**Without adjustment**	With adjustment*
<25	<85	865 236	5 634	7 923 970.41	0.71	1 (Ref.)	1 (Ref.)
<25	≥85	19 498	241	177 082.75	1.36	1.92 (1.68,2.18)	1.3 (1.14,1.48)
≥25	<85	160 340	1 380	1 461 898.97	0.94	1.33 (1.25,1.41)	1.15 (1.08,1.22)
≥25	≥85	111 100	1 346	1 008 796.13	1.33	1.88 (1.77,1.99)	1.41 (1.32,1.5)
Postmenopause							
**BMI**	**WC**	**N**	**Event**	**Duration**	**Rate**	**Without adjustment**	With adjustment*
<25	<85	776 963	56 344	6 506 233.24	8.66	1 (Ref.)	1 (Ref.)
<25	≥85	79 426	9 502	630 666.15	15.07	1.75 (1.71,1.78)	1.17 (1.14,1.19)
≥25	<85	207 544	14 143	1 766 075.61	8.01	0.92 (0.91,0.94)	0.98 (0.96,0.999)
≥25	≥85	304 072	28 504	25 15 412.96	11.33	1.31 (1.29,1.33)	1.11 (1.09,1.13)

BMI, body mass index; WC waist circumference

*Adjustment for age, smoking status, alcohol intake, income, regular exercise, diabetes mellitus, hypertension, dyslipidemia, rheumatic arthritis, hyperthyroidism, chronic kidney disease, chronic obstructive pulmonary disease, hypopituitarism, hyperparathyroidism, Cushing’s syndrome, hyperprolactinemia, vitamin D deficiency, hypercalciuria, intestinal malabsorption, chronic liver disease, anorexia nervosa, systemic lupus erythematosus, irritable bowel disease, secondary amenorrhea, convulsions, depression, use of tricyclic and tetracyclic antidepressants, panic disorder, anxiety disorder, and use of bisphosphonates.

## Discussion

In this nationwide study, we elucidated the association between obesity and vertebral fracture in premenopausal and postmenopausal women. We have found that participants with high BMI, WC, or WHtR had a higher risk of vertebral fracture. In obese women with a BMI over 25 kg/m^2^, abdominal obesity was strongly associated with vertebral fracture risk in the pre and postmenopausal groups. Furthermore, this association was stronger in premenopausal women.

These findings have important implications. First, obesity is not a protective factor against vertebral fracture. Obesity itself should not be regarded as a major risk factor for vertebral fracture. Rather, abdominal obesity should be regarded as a significant factor. Second, BMI and abdominal obesity should be used in combination in strategies for the prevention of vertebral fracture. Lastly, although the results indicated that abdominal obesity could deteriorate bone health in postmenopausal women, premenopausal women showed a stronger association between abdominal obesity and vertebral fracture risk. Thus, advice or intervention concerning body weight and fracture should not be limited to postmenopausal women.

The association between weight fluctuation and fracture risk remains controversial. Two cross-sectional studies showed a positive association between BMI and vertebral fracture in postmenopausal women [26, 27], while a study on 3,683 women aged over 75 across the United States showed that participants who lost weight had higher fracture risk and participants who gained weight had lower fracture risk [28]. A large prospective study by Prieto-Alhambra et al. found no association between BMI and vertebral fracture [10]. However, such results cannot be applied to the Korean population, in which the average BMI is considerably lower.

Obesity has various effects on bone health. The increase in body weight stimulates bone formation, increasing BMD and tissue padding and lessening the impact of a fall and protecting against fractures. However, there are also some negative effects. According to the SWAN study, a higher BMI was associated with greater BMD. However, in a study on femoral neck strength in individuals with obesity, increased BMD with a high mechanical load could not compensate for the high impact force of the fall. After adjustment for BMD, greater BMI was associated with an increased risk of a femoral neck fracture [29].

This study has the strength of being based on nationwide, large-scale data. Furthermore, only a few studies have included both premenopausal and postmenopausal women to assess the menopause-based differences in the association between obesity and BMD; in contrast, we investigated these effects separately in premenopausal and postmenopausal women and obtained comprehensive data that can be readily applied in clinical practice.

Nevertheless, our study has a few limitations. First, due to the nature of retrospective design using self-reported questionnaires, data may be subjected to recall bias. This study was cross-sectional in nature; thus, the causal relationship between obesity and the risk of vertebral fracture could not be explained. Furthermore, a future prospective study would be able to clarify this causal relationship based the results of this study. Second, in previous studies, the association between BMI and fracture risk was established, and it was found to be site-specific. In this study, we have focused on vertebral fractures; however, further studies on different fracture sites are required to navigate additional associations. Last, there are few other characteristics known to be associated with the risk of vertebral fractures in menopausal women, which cannot be neglected. Low education was associated with a greater incidence of fracture in non-white women [30]. Low socioeconomic status is associated with obesity and increased prevalence of chronic diseases, and this has been linked to increased fracture risk [31]. Treatment rates in postmenopausal women are low, and women with obesity showed particularly low treatment rates. According to The Global Longitudinal Study of Osteoporosis in Women, in the 2-year follow-up period after incident fracture, only 27% of women with obesity received bone-protection treatment, compared with 41% of women without obesity and 57% of underweight women [32]. Overall, these findings reveal the pleiotropic effects of obesity on fracture risk regarding the socioeconomic status and bone health during menopause.

These findings can be used to encourage pre and postmenopausal women with obesity to reduce their WC and to highlight the importance of early intervention for obesity, especially abdominal obesity, to prevent vertebral fractures in subsequent years.

## Conclusions

We examined the association between body composite indices of obesity and the risk of vertebral fracture among pre and postmenopausal women and found that obesity and abdominal obesity were associated with a higher risk of vertebral fracture in both pre and postmenopausal women; however, the association was stronger in premenopausal women. Additionally, abdominal obesity significantly contributes to the development of vertebral fractures. We postulate that weight control can prevent vertebral fractures, particularly in premenopausal women. Further studies are required to clarify whether the management of obesity and abdominal obesity can prevent vertebral fractures.
